# All women with multiple sclerosis should start hormone replacement therapy at menopause unless contraindicated: Yes

**DOI:** 10.1177/13524585241255002

**Published:** 2024-06-22

**Authors:** Rhonda Voskuhl

**Affiliations:** UCLA Department of Neurology, UCLA Multiple Sclerosis Program, UCLA Comprehensive Menopause Care Program, University of California, Los Angeles (UCLA), Los Angeles, CA, USA

Since multiple sclerosis (MS) is an autoimmune and neurodegenerative disease, the answer to this question must be consistent with known sex differences in autoimmune and neurodegenerative diseases. Healthy women and men show differences in immune responses and brain subregion physiology.^1,2^ The Genotype-Tissue Expression Project reported that 37% of all genes exhibit sex-biased expression in at least one tissue.^3^ Ubiquitous sex differences can be due to sex hormones, sex chromosomes, or both. In healthy women, menopause induces cognitive domain-specific deficits and accelerates brain region-specific abnormalities on magnetic resonance imaging (MRI), with hippocampus and prefrontal cortex most vulnerable. Longer durations of menopause, either natural or surgical, correlate with worse neuroimaging changes years later. Thus, cognitive issues of menopause are not merely due to temporary hot flashes and poor sleep; they align with structural and functional changes in brain subregions.^2^ Since neuroprotective effects of ovarian hormones are lost during menopause, concluding not to pursue hormone treatment to prevent this quite frankly defies logic. Were past hormone replacement therapy (HRT) regimens designed to achieve brain region-specific protection while minimizing off-target side effects? That is a different question, one not to be conflated with the original question. The loss of neuroprotective effects of ovarian hormones during menopause should be prevented, especially in menopausal women with MS.

Autoimmune diseases, including MS, are more frequent in women than men. X chromosome genes can exert proinflammatory properties by altering expression of autosomal genes through epigenetic regulation and other mechanisms.^1^ Evolutionary pressures drive sex chromosomes and sex hormones toward achieving the most optimal balance for survival of each sex at critical life stages. Sex hormones, namely estrogens, have neuroprotective properties mediated by binding estrogen receptors (ERs) in brain in models of menopause and MS.^2^ Thus, XX genotype can be disease promoting through its effect on the immune system, while estrogen can be disease protective through its effect on the brain.

Notably, testosterone can be converted to estrogen by aromatase in brain. Despite females being more susceptible to MS, males have shown worse disability progression and more regional brain atrophy, when assessed at mean ages of early 40s. This is an age after the beginning of the gradual decline in testosterone in men (1%–2% per year starting at age 30 years) and before the relatively abrupt and complete loss of estrogen in women (around age 50 years).^1,2^ This counterintuitive observation of increased MS susceptibility in females, but worse MS disability progression in males, reveals differential effects of sex-related factors on susceptibility (XX) versus disability (estrogen).

Deleterious effects of andropause and menopause on MS disability progression, albeit with differential timing, prompts consideration of another clinical observation. What is the effect of aging on MS? Aging does not increase relapses or gadolinium-enhancing, inflammatory lesions. Instead, it worsens disability and neurodegeneration. While disease duration was thought to cause the transition from relapsing remitting MS to secondary progressive MS, it now appears that reaching an age of midlife (around age 50 years) contributes to worsening of disability progression, with most MS studies including more women than men. Aging entails many processes, several of which may not be preventable. Focus is needed on aging processes that may be preventable, namely the loss of sex hormones.^1,2^ Treatment durations for clinical trials in women would be feasible, since menopause occurs over 1–2 years at mean age 51 years.

Data from questionnaires revealed that subjective MS symptoms worsen during menopause. Objective data from cohorts of MS women followed longitudinally using standardized neurological exams demonstrated disability worsening at menopause. Cerebral cortex atrophy correlated with an early marker of ovarian failure, namely anti-Mullerian hormone levels.^1,2^ Together, this suggested that menopause worsens disability and neurodegeneration in women with MS.

Why not merely treat MS menopausal women with standard HRT? It was not designed for neuroprotection.^2^ Primary outcomes in HRT trials in healthy menopausal women examined potential benefit on cardiovascular disease, osteoporosis, or hot flashes. These trials were neither proposed nor powered to determine an effect on cognitive domain-specific deficits of verbal memory or processing speed. Instead, exploratory outcomes included assessment for the presence of global dementia, with the type of dementia unclear. When brain imaging was done, it usually entailed global brain changes, not region-specific abnormalities in cerebral cortex or hippocampus. The past HRT key was not designed to unlock neurodegeneration.

The type and dose of estrogen is critical to achieve efficacy in brain and minimize toxicity elsewhere. There is no evidence that the various low-dose estrogens in Premarin will provide neuroprotection. Estradiol could be neuroprotective based on preclinical data and short-term cognitive benefits in surgical menopause patients. However, estradiol cannot be used long term to prevent neurodegeneration because it binds strongly to ERa, the receptor in breast associated with cancer risk. Estradiol’s use is limited to the lowest possible dose and duration, usually temporarily for hot flashes. To provide brain protection and avoid off-target effects on breast, ERb ligands are being investigated.^2^ A menopause model showed that ligation of ERb on astrocytes improved cognitive testing performance and reduced hippocampal atrophy, while decreasing glial activation and synaptic loss.^2^ In MS models, ERb ligand treatment decreased microglial activation, reduced synaptic loss, and induced remyelination through binding to microglia and oligodendrocytes.^4,5^

Estriol, the pregnancy estrogen, has been used in Europe for decades at a 2-mg dose for alleviation of menopausal symptoms in healthy women. It binds ERa weakly, acting mainly through ERb. Estriol treatment in the MS model reduced hippocampal atrophy and cerebral cortex atrophy, while decreasing neuropathology therein.^6,7^ Studies of estriol treatment in MS women using an 8-mg dose to induce mid-pregnancy serum levels demonstrated improved cognitive processing speed, reduced cerebral cortex atrophy, and decreased serum neurofilament light chain levels, each as compared to placebo treatment and within 12 months.^8–10^

In addition to estrogen type, dose and compliance matter. Improvement in cognitive testing correlated with higher estriol blood levels.^10^ Consideration of estriol treatment in menopausal women with MS is now warranted using disability-specific clinical outcomes and brain region-specific imaging biomarkers.
